# Inhibition of autophagy promotes metastasis and glycolysis by inducing ROS in gastric cancer cells

**DOI:** 10.18632/oncotarget.5674

**Published:** 2015-10-14

**Authors:** Wenjie Qin, Chao Li, Wen Zheng, Qingqu Guo, Yuefeng Zhang, Muxing Kang, Bo Zhang, Bin Yang, Baozhong Li, Haijun Yang, Yulian Wu

**Affiliations:** ^1^ Department of Surgery, Second Affiliated Hospital, Zhejiang University School of Medicine, Hangzhou, P. R. China; ^2^ Key Laboratory of Cancer Prevention and Intervention, China National Ministry of Education, Cancer Institute, Second Affiliated Hospital, Zhejiang University School of Medicine, Hangzhou, Zhejiang, P. R. China; ^3^ Department of Oncosurgery, Anyang Tumor Hospital, Henan, P. R. China; ^4^ Department of Pathology, Anyang Tumor Hospital, Henan, P. R. China

**Keywords:** autophagy inhibition, metastasis, glycolysis, reactive oxygen species (ROS), antioxidant

## Abstract

Autophagy defect has been shown to be correlated with malignant phenotype and poor prognosis of human cancers, however, the detailed mechanisms remain obscure. In this study, we investigated the biological changes induced by autophagy inhibition in gastric cancer. We showed that inhibition of autophagy in gastric cancer cells promotes epithelial-mesenchymal transition (EMT) and metastasis, alters metabolic phenotype from mitochondrial oxidative phosphorylation to aerobic glycolysis and converts cell phenotype toward malignant, which maybe further contribute to chemoresistance and poor prognosis of gastric cancer. We also identified that the EMT and metabolism alterations induced by autophagy inhibition were dependent on ROS-NF-κB-HIF-1α pathway. More importantly, scavenging of ROS by the antioxidant N-acetylcysteine (NAC) attenuated activation of NF-κB and HIF-1α in autophagy-deficient gastric cancer cells, and autophagy inhibition induced metastasis and glycolysis were also diminished by NAC *in vivo*. Taken together, our findings suggested that autophagy defect promotes metastasis and glycolysis of gastric cancer, and antioxidants could be used to improve disease outcome for gastric cancer patients with autophagy defect.

## INTRODUCTION

Gastric cancer is the second deadliest disease among cancers worldwide and the incidence rate is highest in Eastern Asia [1–3]. Although therapeutic strategies are continually improved, overall 5-year survival is still less than 30% [4]. Thus, it's urgent to explore for more effective treatment options. Due to the heterogeneity of gastric cancer, there are individual differences in tumor aggressiveness, histopathologic features, and treatment response [5]. Therefore, identifying specific biomarkers may facilitate the selection of patients to provide individualized therapy.

Autophagy is a dynamic catabolic process to maintain intracellular homeostasis. Autophagy degrades intracellular damaged proteins and organelles and converts them to metabolic intermediates in the autolysosomes for energy generation and biosynthesis [6, 7]. Autophagy can support tumor cells survival through generating substrates for mitochondrial metabolism, preventing metabolic stress, mitigating accumulation of toxic substances and so on [7, 8]. On the other hand, defective autophagy is involved in the onset and progression of tumors via inducing inflammation and necrosis [9–11]. Thus, the role of autophagy in tumors is complicated and further investigation is needed to elucidate the character of autophagy.

The expression of BECN1, an important autophagy initiator, is suppressed in several human tumors, such as ovarian, breast and prostate cancers [12]. Recently, BECN1 has been reported to be positively correlated with tumor differentiation in several cancers, and can be used as an independent biomarker for predicting overall survival and progression-free survival in gastric and liver cancer patients [12–15]. These studies have illustrated that autophagy defect may be associated with malignant phenotype and poor prognosis of human cancers, however, the specific mechanisms are still unclear. In this study, we gained further insights into the role of autophagy in gastric cancer progression. Our results indicated that autophagy provides metabolic intermediates for mitochondrial oxidative phosphorylation to fuel cell survival, while autophagy defect causes the shift from oxidative phosphorylation to aerobic glycolysis for proper energy homeostasis in gastric cancer cells. Besides, we also found that autophagy defect induces other biological changes, including promoting epithelial-mesenchymal transition (EMT), metastasis and malignant transformation. Importantly, ROS generation is involved in the autophagy defect induced biological changes, and scavenging of ROS by the antioxidant N-acetylcysteine (NAC) could revert these biological changes in gastric cancer cells.

## RESULTS

### Autophagy inhibition promotes EMT of gastric cancer cells through HIF-1α activation

**Figure 1:**
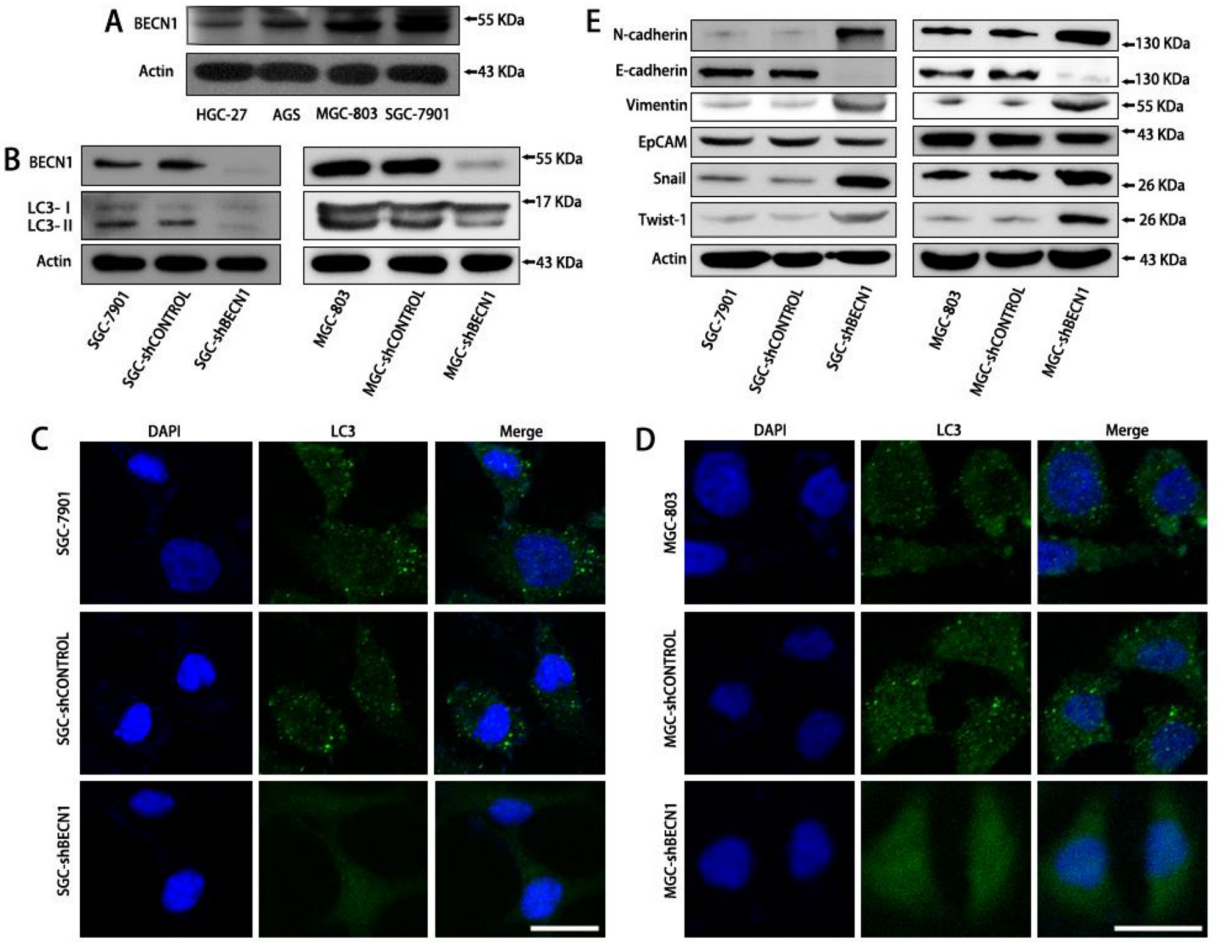
Autophagy inhibition promotes EMT of SGC-7901 and MGC-803 cells **A.** The BECN1 expression in four human gastric cancer cell lines (AGS, HGC-27, MGC-803 and SGC-7901) was determined by Western blot analysis. **B–D.** SGC-7901 and MGC-803 cells with stable autophagy inhibition (SGC-shBECN1 and MGC-shBECN1 cells) were established through knockdown of BECN1 by lentiviral transduction. (B) SGC-shBECN1 and MGC-shBECN1 cells displayed decreased expression of BECN1 and LC3-II compared with control cells by Western blot analysis. (C & D) immunofluorescence analysis showed that LC3 protein was distributed in a diffused pattern in SGC-shBECN1 and MGC-shBECN1 cells, whereas LC3 protein was localized in autophagic vacuoles (dot-like structures) in control cells. Scale bar, 20 μm. **E.** EMT markers (N-cadherin, E-cadherin, Vimentin, EpCAM, Snail and Twist-1) expression were determined in SGC-7901 and MGC-803 cells with or without autophagy inhibition by Western blot analysis.

The expression of BECN1 was evaluated in four human gastric cancer cell lines (AGS, HGC-27, MGC-803 and SGC-7901). As shown in Figure 1A, BECN1 expression was higher in SGC-7901 cells (moderately differentiated cells) than that in AGS cells (poorly differentiated cells), MGC-803 cells (poorly differentiated cells) and HGC-27 cells (undifferentiated cells). SGC-7901 and MGC-803 cells with stable autophagy inhibition (SGC-shBECN1 and MGC-shBECN1 cells) were generated through knockdown of BECN1 by lentiviral transduction (Figure 1B–1D). Except EpCAM, autophagy inhibition increased the expression of mesenchymal markers such as N-cadherin, Vimentin, Snail, Twist-1 in SGC-7901 and MGC-803 cells (Figure 1E). In contrast, the expression of E-cadherin, an epithelial marker, was suppressed in SGC-shBECN1 and MGC-shBECN1 cells compared with control cells (Figure 1E). Consistent with these results, the morphology of SGC-7901 and MGC-803 cells became more spindle-like and scattered after autophagy inhibition (Supplementary Figure S1).

**Figure 2:**
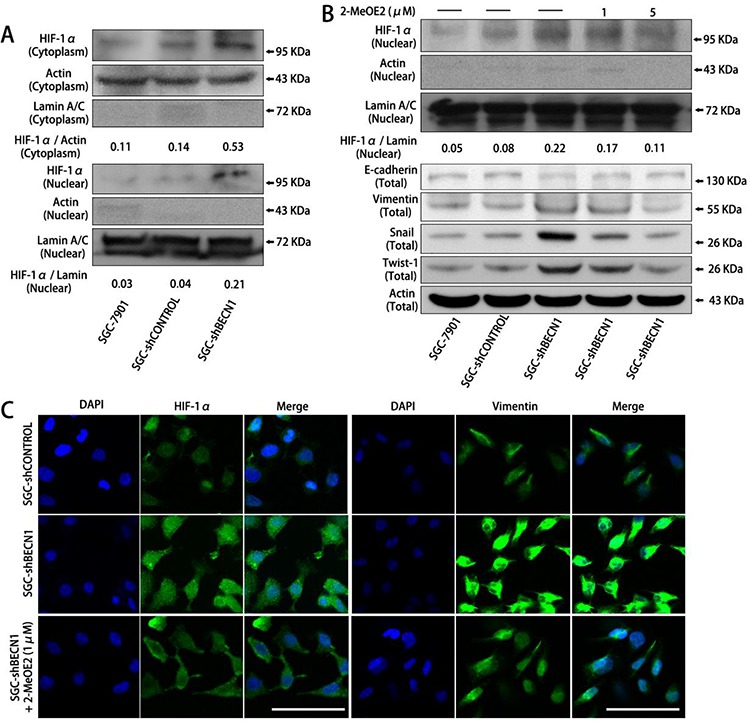
The EMT induced by autophagy inhibition is dependent on HIF-1α activation in gastric cancer cells **A.** In normoxia, HIF-1α expression in cytoplasm and nucleus of SGC-shBECN1 and control cells were analyzed by Western blot. The density ratio of HIF-1α / Actin in cytoplasm and HIF-1α / Lamin in nucleus were calculated to quantify the Western blot signals. **B & C.** After pretreatment with 1 or 5 μM 2-MeOE2 for 48 h, nuclear translocation of HIF-1α and expression of EMT markers in SGC-shBECN1 cells were determined by Western blot analysis or immunofluorescence analysis respectively. Scale bar, 100 μm.

Furthermore, we found that autophagy defect promoted intracellular accumulation and nuclear localization of HIF-1α in SGC-7901 and MGC-803 cells under normoxic circumstance (Figure 2A & 2C left; Supplementary Figure S3B). Nevertheless, the HIF-2α expression was not affected by autophagy inhibition (Supplementary Figure S2A). To investigate whether EMT induced by autophagy inhibition was dependent on HIF-1α activation, SGC-shBECN1 cells were treated with 1 μM and 5 μM 2-MeOE2 (a specific inhibitor of HIF-1α) for 48 h. As shown in Figure 2B and 2C, the HIF-1α nuclear translocation and EMT induced by autophagy inhibition were reverted by 2-MeOE2. These results were further verified by HIF-1α siRNA in SGC-shBECN1 cells (Supplementary Figure S2B & S2C). Altogether, these experiments demonstrated that compromised autophagy could enhance the EMT phenotype of gastric cancer cells and this process could be HIF-1α-dependent.

**Figure 3:**
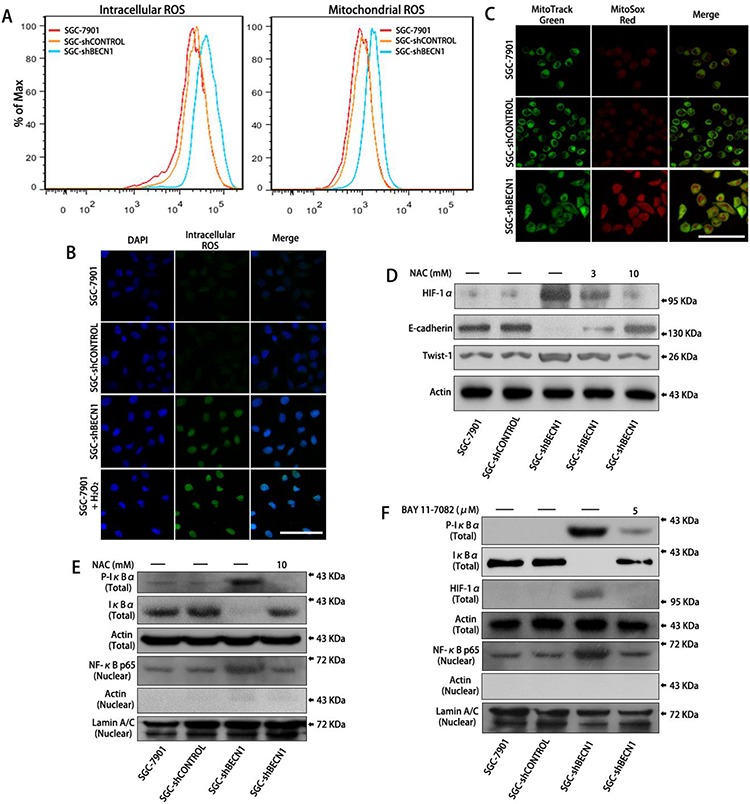
Autophagy defect in gastric cancer cells facilitates HIF-1α expression by ROS-NF-κB-HIF-1α pathway **A.** Intracellular ROS level was determined after pretreatment with DCF-DA (left panel) and mitochondrial ROS level was determined after pretreatment with MitoSox Red (right panel) by flow cytometry (FCM) analysis respectively. **B & C.** Intracellular and mitochondrial ROS levels were observed by a fluorescence confocal microscope. Scale bar, 100 μm. **D.** SGC-shBECN1 cells were treated with 3 or 10 mM NAC for 48 h, then the expression of HIF-1α and EMT markers were determined by Western blot. **E.** After incubation with or without 10 mM NAC for 48 h, the expression of Phospho-IκBα and IκBα in whole cells and the expression of NF-κB p65 in nuclei were analyzed by Western blot. **F.** After pretreatment with or without 5 μM BAY 11–7082 for 24 h, the expression of Phospho-IκBα, IκBα, HIF-1α in whole cells, and the expression of NF-κB p65 in nuclei were determined by Western blot.

### Autophagy defect increases HIF-1α expression by ROS-NF-κB-HIF-1α pathway in gastric cancer cells

Previous studies have demonstrated that autophagy defect results in an increase in intracellular ROS level as well as mitochondrial ROS level [16, 17]. Consistent with these studies, our results showed that both intracellular and mitochondrial ROS levels were increased in SGC-shBECN1 and MGC-shBECN1 cells compared with control cells (Figure 3A–3C & Supplementary Figure S3A). Then, we tested whether activation of HIF-1α was associated with increased ROS in autophagy deficient cells. After incubating SGC-shBECN1 and MGC-shBECN1 cells with 3 mM and 10 mM NAC (ROS scavenging agent) for 48 h, the HIF-1α accumulation was down-regulated and the EMT was reverted (Figure 3D & Supplementary Figure S3B), suggested that autophagy defect induced HIF-1α activation and EMT are based on ROS generation.

We also found that loss of autophagy in gastric cancer cells led to phosphorylation and degradation of IκBα, facilitated nuclear translocation of NF-κB, moreover, these changes were suppressed by NAC (Figure 3E & Supplementary Figure S3B). According to previous report [18], these results suggested that increased ROS caused by autophagy inhibition activates NF-κB through the classical IKK-dependent pathway. Afterwards, we tested whether NF-κB activation was involved in the ROS-induced accumulation of HIF-1α. After treating SGC-shBECN1 cells with 5 μM BAY 11–7082 (a NF-κB inhibitor via restraining phosphorylation and degradation of IκBα) for 24 h, the induced nuclear translocation of NF-κB and overexpression of HIF-1α were attenuated (Figure 3F). Similarly, the HIF-1α upregulation was inhibited by NF-κB p65 siRNA in SGC-shBECN1 cells (Supplementary Figure S3C & S3D). Since HIF-1α gene has been reported as a transcriptional target of NF-κB [19, 20], we performed EMSA to evaluate the autophagy defect mediated transcriptional activity of NF-κB in gastric cancer cells using nuclear extracts and specific probe (containing NF-κB binding site in HIF-1α promoter). The protein-probe binding was more intensive with the loading protein derived from SGC-shBECN1 cells than control cells, and NAC could abrogate the NF-κB-probe binding (Supplementary Figure S3E). In summary, these results indicated that increased expression of HIF-1α induced by autophagy defect is based at least in part on ROS-NF-κB-HIF-1α pathway.

### Autophagy inhibition alters metabolic phenotype of gastric cancer cells

**Figure 4:**
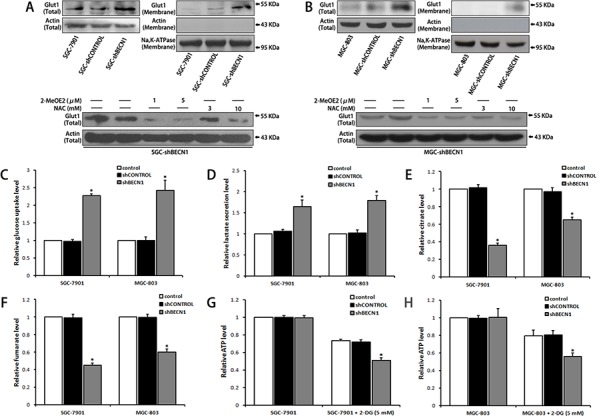
Autophagy inhibition alters metabolic phenotype of gastric cancer cells **A & B.** Glut1 expression in whole cells and cytomembranes were determined by Western blot (upper panel). SGC-shBECN1 and MGC-shBECN1 cells were treated with 2-MeOE2 (1 or 5 μM) or NAC (3 or 10 mM) and then Glut1 expression was determined by Western blot (lower panel). **C & D.** Glucose uptake and lactate secretion levels of autophagy-deficient cells and control cells were measured by colorimetric analysis. **E & F.** Intracellular citrate and fumarate levels were measured by colorimetric analysis. **G & H.** After incubation with or without 5 mM 2-DG for 12 h, intracellular ATP levels were measured by colorimetric analysis. Each column represents the average of 3 independent experiments. Bars represent mean ± SD. *: *P* < 0.05.

Because HIF-1α is a key regulator of cell metabolism [21], we tested whether autophagy inhibition could influence cell’ metabolism. As shown in Figure 4A and 4B, autophagy defect enhanced total expression of glucose transporter 1 (GLUT-1) and promoted membrane translocation of GLUT-1 in gastric cancer cells, the enhanced GLUT-1 expression could be reverted by 2-MeOE2 or NAC. Correspondingly, the glucose uptake level of SGC-shBECN1 and MGC-shBECN1 cells was increased significantly compared with control cells (Figure 4C), meanwhile, the lactate secretion level of these cells was also increased (Figure 4D), implying that autophagy defect induces aerobic glycolysis in gastric cancer cells. On the other hand, the concentrations of citrate and fumarate, two intermediates in the TCA cycle (tricarboxylic acid cycle) used to form ATP, were decreased in SGC-shBECN1 and MGC-shBECN1 cells (Figure 4E & 4F), indicating that autophagy defect reduces mitochondrial oxidative phosphorylation level in gastric cancer cells. However, there was no significant difference of ATP production between autophagy deficient cells and control cells (Figure 4G & 4H). To investigate whether ATP generation could be affected by autophagy defect induced glycolysis, all cells were treated with 5 mM 2-DG (2-Deoxy-D-glucose, an inhibitor of glycolysis) for 12 h. Our results displayed that ATP generation of all cells was inhibited by 2-DG and the inhibitory effect was more significant in SGC-shBECN1 and MGC-shBECN1 cells than control cells (Figure 4G & 4H), indicating that autophagy deficient cells are more relied on glycolysis to provide energy and the decreased ATP production in mitochondria is compensated by increased glycolysis in autophagy deficient cells. Besides, the EMT couldn't be reverted by 2-DG in SGC-shBECN1 and MGC-shBECN1 cells (Supplementary Figure S4), demonstrating that autophagy inhibition induced glycolysis has no appreciable effect on EMT. Altogether, these results suggested that autophagy defect causes the shift from oxidative phosphorylation to glycolysis in gastric cancer cells for maintaining proper energy homeostasis.

### Autophagy inhibition facilitates malignant transformation of gastric cancer cells

**Figure 5:**
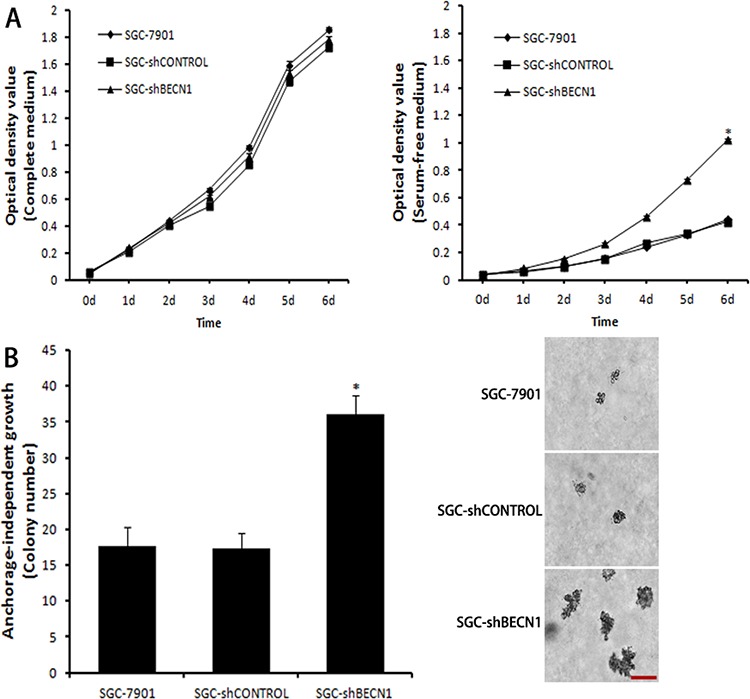
Autophagy inhibition facilitates malignant transformation of gastric cancer cells **A.** Under complete medium culture condition (left panel) or serum-free condition (right panel), cell proliferation was measured by MTT assay. **B.** Anchorage-independent growth was assessed by soft agar assay. The colonies were counted and the sizes of these colonies were observed by a microscope. Scale bar, 100 μm. Each point or column represents the average of 3 independent experiments. Bars represent mean ± SD. *: *P* < 0.05.

Next, we assessed whether autophagy inhibition could increase the malignant level of gastric cancer cells. Under the complete medium culture condition, proliferation of SGC-shBECN1 cells was similar to parental cells (Figure 5A left). Even though the growth of all cells was suppressed under serum-free condition, SGC-shBECN1 cells grew significantly faster than control cells (Figure 5A right), suggesting that SGC-shBECN1 cells acquired the ability of serum-independent growth partially. Then, we determined the anchorage-independent growth of these cells by soft agar assay. As shown in Figure 5B, the colonies number of SGC-shBECN1 cells was higher and the sizes of these colonies were larger than control cells. Since serum-independent proliferation and anchorage-independent growth are positively correlated with the degree of tumor malignancy, these data indicated that loss of autophagy enhances malignant transformation of gastric cancer cells.

### Autophagy defect promotes metastasis and glycolysis of gastric cancer cells *in vivo*, and NAC restrains these effects

**Figure 6:**
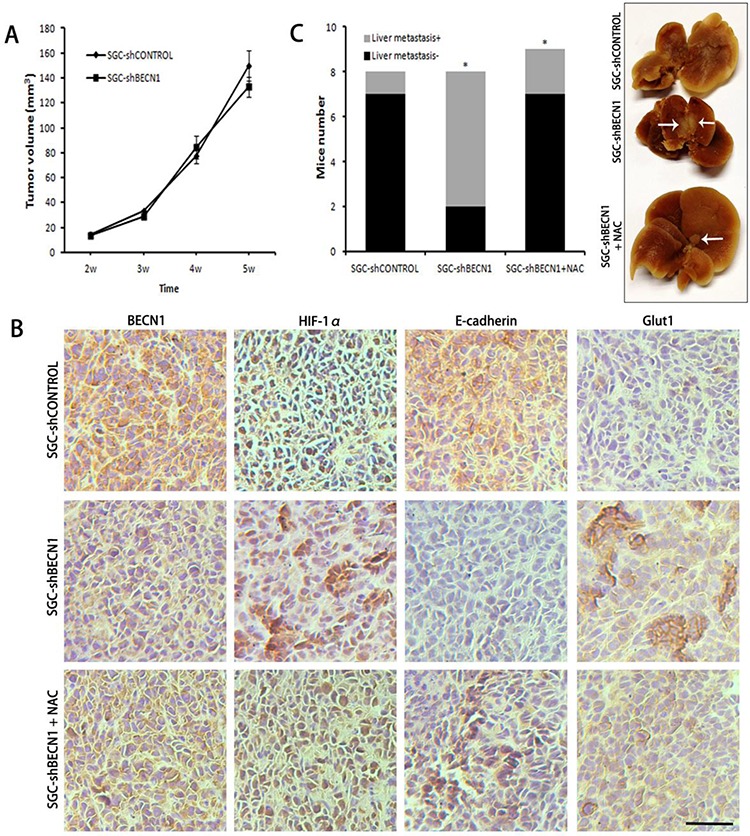
Antioxidant NAC reverts autophagy inhibition induced metastasis *in vivo* **A.** The subcutaneous tumors were established in nude mice by implanting SGC-shCONTROL and SGC-shBECN1 cells. Tumor masses of all mice were measured twice a week and tumor volume was calculated. **B.** The expression of BECN1, HIF-1α, E-cadherin and Glut1 in xenografts from SGC-shCONTROL, SGC-shBECN1 and SGC-shBECN1 + NAC groups were determined by Immunohistochemical analysis. Scale bar, 100 μm. **C.** Liver metastatic mice models were induced by splenic inoculation of SGC-shCONTROL and SGC-shBECN1 cells. The liver metastatic mice of three groups were counted respectively. The white arrows pointed to metastatic nodules in livers. Bars represent mean ± SD. *: *P* < 0.05.

**Figure 7:**
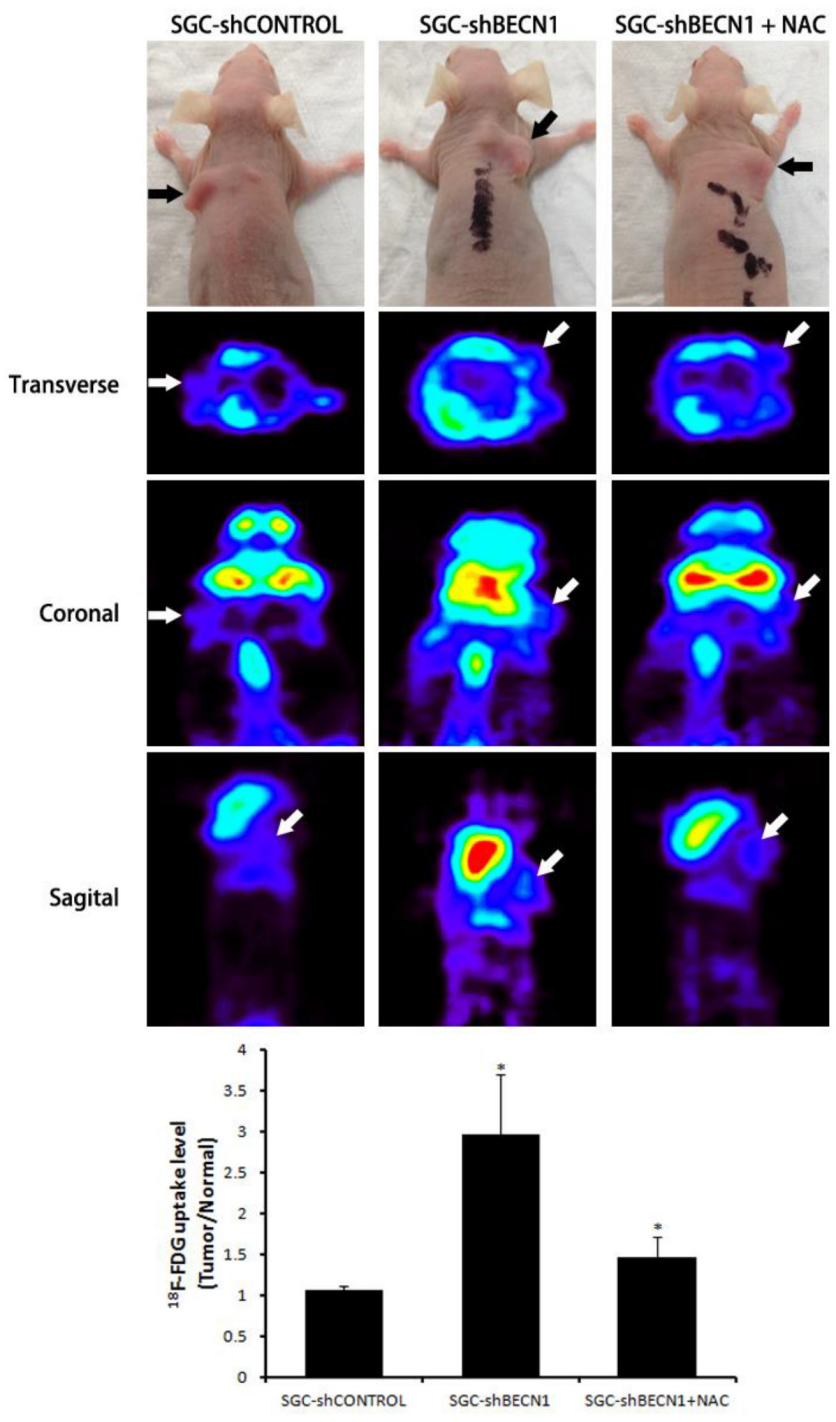
Antioxidant NAC restrains autophagy inhibition induced glycolysis *in vivo* The glucose uptake in subcutaneous xenografts from SGC-shCONTROL, SGC-shBECN1 and SGC-shBECN1 + NAC groups were detected by microPET using ^18^F-FDG. ^18^F-FDG uptake values in xenografts were calculated using the formula: Tumor / Normal ratio = pixel maximum uptake in tumor / pixel mean uptake in upper limb muscles. The black and white arrows pointed to subcutaneous xenografts. Bars represent mean ± SD. *: *P* < 0.05.

The subcutaneous tumor models in nude mice were established by implanted SGC-shCONTROL and SGC-shBECN1 cells respectively. Autophagy defect did not have a significant effect on the growth of xenografts (Figure 6A). Immunohistological staining showed that expression of HIF-1α and GLUT-1 were up-regulated in SGC-shBECN1 group compared to SGC-shCONTROL group, and the expression of E-cadherin was just the opposite (Figure 6B). Based on these results, we examined whether autophagy defect could promote metastasis of gastric cancer cells firstly. Liver metastatic mice models were induced by splenic inoculation of SGC-shCONTROL and SGC-shBECN1 cells, and six mice (6/8) in SGC-shBECN1 group formed visible metastases in livers compared to one mouse (1/8) in SGC-shCONTROL group (*P* < 0.05) (Figure 6C). Next, we detected glucose uptake in subcutaneous xenografts by PET using ^18^F-FDG (an analog of glucose). ^18^F-FDG uptake of xenografts was more obvious in SGC-shBECN1 group than SGC-shCONTROL group (Figure 7), which further confirmed that autophagy inhibition promotes glycolysis in gastric cancer cells. Furthermore, administration of NAC could suppress these biological changes induced by autophagy inhibition effectively *in vivo* (Figure 6B & 6C; Figure 7).

### Correlation between BECN1 expression and clinicopathologic features in gastric cancer patients

**Figure 8:**
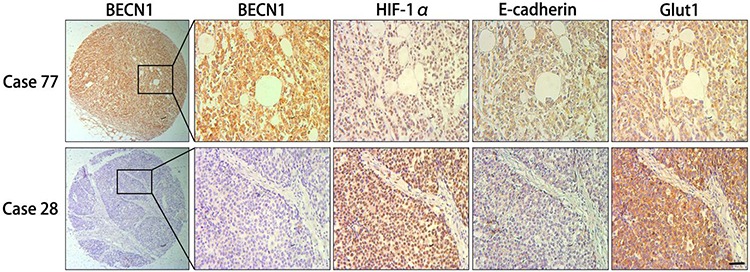
Immunohistochemical analysis of consecutive sections from human gastric cancer tissues First row, the expression of HIF-1α, E-cadherin and Glut1 in positive BECN1 expression case (case 77). Second row, the expression of HIF-1α, E-cadherin and Glut1 in negative BECN1 expression case (case 28). Scale bar, 100 μm.

In this study, Tissue microarrays (TMAs) consisting of human gastric cancer tissues from 156 patients were used to research the correlation between autophagy and clinical features of gastric cancer. According to BECN1 expression in these tissues, 156 cases were divided into high expression group (58, 37.2%) and low expression group (98, 62.8%). Consistent with previous study [12], negative expression of BECN1 was positively associated with poorer histology differentiation (*P* = 0.020, Table 1) and more advanced clinical stage (*P* < 0.001, Table 1). Compared with patients in the group with high expression of BECN1, patients in the low expression group were more likely to accompany with deeper tumor invasion (*P* = 0.074, Table 1) and lymph node metastasis (*P* = 0.061, Table 1), although these differences were not statistically significant. Moreover, BECN1 expression was negatively correlated with expression of HIF-1α (*P* = 0.042) and GLUT-1 (*P* = 0.046), and positively correlated with E-cadherin expression (*P* = 0.006) in gastric cancer tissues (Table 1; Figure 8 & Supplementary Figure S5). There was no significant correlation between BECN1 expression and age or gender of gastric cancer patients.

**Table 1:** Correlation between BECN1 expression and clinicopathological features in gastric cancer patients

Parameter	Total(*n* = 156)	BECN1		*P*-value^a^
		High expression	Low expression	
**Age (years)**				
< 60	61	25	36	0.498
≥ 60	95	33	62	
**Gender**				
Male	119	46	73	0.562
Female	37	12	25	
**Clinical stage**				
I + II	54	34	20	0.000
III + IV	102	24	78	
**Tumor differentiation**				
Well	80	37	43	0.020
Poorly	76	21	55	
**Depth of invasion**				
T_1_ + T_2_	26	14	12	0.074
T_3_ + T_4_	130	44	86	
**Nodal metastasis**				
N_0_ + N_1_/ No	42	21	21	0.061
N_2_ + N_3_/ Yes	114	37	77	
**HIF-1α**				
High expression	97	30	67	0.042
Low expression	59	28	31	
**E-cadherin**				
High expression	56	29	27	0.006
Low expression	100	29	71	
**GLUT1**				
High expression	89	27	62	0.046
Low expression	67	31	36	

a *P*-values were calculated using Chi-square test.

## DISCUSSION

In the 1920s, Otto Warburg put forward the claim that the energy supply of cancer cells mainly rely on aerobic glycolysis, which is in contrast to normal cells [22]. Because autophagy can provide TCA metabolites and contribute to ATP generation [7], the role of autophagy in energy metabolism of gastric cancer cells was examined in this study. We discovered that inhibition of autophagy in gastric cancer cells reduced the production of citrate and fumarate, promoted the expression and membrane translocation of GLUT-1 as a result of the increased HIF-1α expression, consequently enhanced the glucose uptake and lactate production. Thus, our results indicated that autophagy defect induces the metabolic alteration from oxidative phosphorylation to glycolysis, which is in agreement with previous study [17].

Besides the up-regulation of HIF-1α expression, we also found that autophagy defect could cause an increase in cytoplasmic and mitochondrial ROS levels in gastric cancer cells, which is consistent with previous studies [16, 17]. Recently, many studies reported that HIF-1α accumulation could be regulated by ROS through enhancing transcription of HIF-1α gene and stabilization of HIF-1α protein [23–26]. Indeed, our results showed that HIF-1α accumulation induced by autophagy defect was markedly attenuated by antioxidant NAC. Furthermore, we found that autophagy inhibition facilitated the degradation of IκBα and nuclear translocation of NF-κB, and these processes were reverted by NAC, which is in according with previous report that NF-κB can be activated by ROS through IKK-dependent pathway [18]. Early studies have illustrated that NF-κB is a direct modulator of HIF-1α expression through binding the HIF-1α promoter and initiating the transcription of HIF-1α gene [19, 20]. This is also confirmed in the present study, suggesting that autophagy defect induced nuclear translocation of NF-κB increases HIF-1α mRNA and protein levels in gastric cancer cells. In addition, increased HIF-1α expression can enhance glycolysis via promoting the transcription of glucose transporters and glycolytic enzymes [21, 27]. Therefore, our results indicated that metabolic alteration of gastric cancer cells induced by autophagy defect could be dependent, at least in part, on ROS-NF-κB-HIF-1α pathway. What's more, immunohistochemical and PET analysis showed that NAC could suppress autophagy defect induced up-regulation of HIF-1α and GLUT-1, as well as glucose uptake in gastric cancer xenografts (Figure 6B & 7), which is further sustaining our hypothesis. However, the relationship between autophagy and cell metabolism is still complex and more studies are needed to elucidate the issue.

Recently, ROS have been verified to be capable of regulating many intracellular signal transduction pathways, and abnormal ROS signal may stimulate carcinogenesis of different cancer cells [23]. Yang et al. have already demonstrated that autophagy defect causes a decrease in oxidative phosphorylation and an increase in ROS level in mitochondrion [17], the similar results were also obtained by us, which seems paradoxical to the fact that mitochondrial ROS production is mainly depend on mitochondrial oxygen metabolism [28, 29]. On the other hand, mitochondrial ROS level could be increased significantly when mitochondrion makes ATP at low level [30], and our results are more likely to support this claim, which may partly explain the mechanism of increased mitochondrial ROS production induced by autophagy inhibition. Currently, the specific mechanism is still unclear and needs more research.

This study also displayed that autophagy defect could induce EMT of gastric cancer cells through up-regulation of HIF-1α expression, in agreement with previous study that EMT regulators are located downstream of HIF-1α [26]. Also, autophagy inhibition promoted hepatic metastasis after splenic implantation of gastric cancer cells in mice. Moreover, the induced EMT and metastasis were suppressed by NAC, which reaffirms the fact that ROS can be involved in tumor metastasis [23, 26]. In summary, our data indicated that autophagy defect also promotes EMT and metastasis of gastric cancer cells via ROS-NF-κB-HIF-1α pathway. Meanwhile, it has been described that autophagy deficiency facilitates EMT of breast cancer cells and embryonic fibroblast cells through inhibiting the degradation of mesenchymal markers such as Snail and Twist-1 [31–34]. Therefore, we concluded that autophagy defect can induce EMT not only through suppressing the degradation of mesenchymal markers but also through increasing the expression of mesenchymal markers via ROS-NF-κB-HIF-1α pathway. However, several studies showed that EMT can be induced by autophagy in pancreatic cancer cells and HK-2 cells [35, 36]. Even though the contradictory results aren't explained in this study, we speculate that the role of autophagy in EMT may vary with different cell types.

Inactivation of autophagy has been shown to correlate with the malignant phenotype of hepatocellular carcinoma [14]. Likewise, we found that autophagy defect facilitated malignant transformation of gastric cancer cells. Consistent with early study [12], our results also demonstrated that autophagy was inhibited in poorly differentiated gastric cancer cells and tissues. Thus, autophagy may decide the differentiation and progression of gastric cancer. However, we didn't find the significant relationship between autophagy defect and growth of gastric cancer cells, it might be due to, although autophagy inhibition can increase HIF-1α expression, HIF-1α has no effect on proliferation and viability of gastric cancer cells [37].

The clinical data and specimens from 156 gastric cancer patients were used in this study. In line with previous research [12], our study showed that low expression of BECN1, an essential autophagy initiator, was associated with advanced clinical stage of gastric cancer patients. And negative expression of BECN1 could be an independent prognostic marker for predicting shorter overall survival and progression-free survival in gastric cancer patients [12, 13]. Moreover, our immunohistochemical analysis demonstrated that BECN1 expression was negatively correlated with HIF-1α and GLUT-1 expression, and positively correlated with E-cadherin expression in human gastric cancer tissues, which indicated that autophagy defect promotes glycolysis and metastasis of gastric cancer in patients. Thus, loss of autophagy may cause the worse prognosis of gastric cancer patients by accelerating the development and progression of this disease. However, how to improve the outcome of these patients still remains a puzzle. The present study suggested that autophagy inhibition induced biological changes, such as metastasis and glycolysis, are ROS dependent in gastric cancer cells, which may be used to provide a novel therapeutic strategy to gastric cancer patients with compromised autophagy. In fact, we found that these biological changes could be reverted effectively by antioxidant NAC via blocking the ROS-NF-κB-HIF-1α pathway. Besides, the inhibitory effect of NAC on these biological changes may also rely on the suppression of mTOR-signaling pathway [38–40] and this issue should be verified by further investigation. Therefore, antioxidants may be valuable for better treatment to gastric cancer patients with autophagy defect.

In conclusion, this study demonstrated that autophagy inhibition promotes glycolysis and metastasis of gastric cancer cells, and these biological changes can be reverted by antioxidant NAC. Therefore, identifying gastric cancer subtypes which autophagic genes are down-regulated may facilitate the selection of patients that will respond sensitively to antioxidants.

## MATERIALS AND METHODS

### Cell culture and reagents

Human gastric cancer cell lines, including AGS, HGC-27, MGC-803 and SGC-7901 were purchased from the cell bank of Chinese academy of sciences and maintained in RPMI-1640 supplemented with 10% fetal calf serum, 100 units/ml penicillin and streptomycin in a humidified atmosphere (5% CO2 and 95% air). 2-Methoxyestradiol (2-MeOE2) and BAY 11–7082 (BAY) were products of Selleck Chemicals (Houston, TX, USA). N-acetylcysteine (NAC) and 2-Deoxy-D-glucose (2-DG) were purchased from Sigma-Aldrich (St. Louis, MO, USA).

### RNA silencing by siRNA and shRNA

Human HIF-1α siRNA (5′-GUAGCCUCUUUGAC AAACUTT-3′) and NF-κB p65 siRNA (5′-GGACAUAU GAGACCUUCAATT-3′) were synthesized by GenePharma (Shanghai, China). The lentiviral vector (pGLVU6/Puro) encoding human BECN1-shRNA (5′-TGCAGTTTGGCACAATCAATATTCA AGAGATATTG ATTGTGCCAAACTGCTTTTTTC-3′) was constructed by GenePharma. Lentiviral vector was cotransfected with packaging vectors (GenePharma) into 293 T cells for packaging. SGC-7901 and MGC-803 cells were infected with lentivirus encoding BECN1-shRNA and scrambled-shRNA respectively. SGC-7901 and MGC-803 cells with stably low expression of BECN1 were termed as SGC-shBECN1 and MGC-shBECN1, SGC-7901 and MGC-803 cells transfected with lentiviral vectors carrying scrambled shRNA were referred as SGC-shCONTROL and MGC-shCONTROL.

### Western blot analysis

Procedures of protein extraction and Immunoblot were described earlier [41]. Nuclear and cytoplasmic proteins were extracted using NE-PER Nuclear and Cytoplasmic Extraction Reagents (Thermo Scientific, Waltham, MA, USA), membrane protein was extracted using Mem-PER Eukaryotic Membrane Protein Extraction Reagent Kit (Thermo Scientific, Waltham, MA, USA). Primary antibodies against beclin-1 (BECN1, ab51031), HIF-1α (ab51608), GLUT-1 (ab115730) and Snail (ab180714) were purchased from Abcam Public Limited Co (Cambridge, MA, USA), antibodies against LC3 (12741), N-cadherin (4061), E-cadherin (3195), Vimentin (5741), EpCAM (3599), IκBα (4814), Phospho-IκBα (2859), NF-κB p65 (8242), HIF-2α (7096), Lamin A/C (4777) and Na, K-ATPase (3010) were products of Cell Signaling Technology, Inc. (CST, Beverly, MA, USA), anti-Twist-1 antibody (AF62301) was purchased from R&D Systems, Inc. (Minneapolis, MN, USA), anti-β-actin antibody (sc-47778) was obtained from Santa Cruz Biotechnology (Dallas, TX, USA).

### Immunofluorescence analysis

Cells were fixed with 4% paraformaldehyde for 15 min at 4°C and blocked with 5% BSA for 1 h at 37°C. Then cells were incubated with primary antibodies against LC3 (CST), HIF-1α (Abcam), Vimentin (CST) respectively at 4°C overnight, followed by incubation for 1 h at room temperature with FITC-conjugated secondary antibody (Abcam, ab150077). Nuclei were stained with DAPI (Sigma). Images were acquired using a confocal microscope (Zeiss, Jena, Germany).

### Intracellular reactive oxygen species (ROS) measurement

Intracellular ROS measurement was performed according to procedures described previously [42]. After treatment with 10 μM DCF-DA (ROS-specific fluorescent probe, Sigma) for 30 min at 37°C, cells were resuspended in ice-cold PBS for flow cytometry (FCM) analysis, or cells stained with DAPI were observed by a confocal microscope. DCF-DA was excited at 488 nm and emitted light was measured at 530 nm (Ex_488 nm_/Em_530 nm_).

### Mitochondrial ROS measurement

Mitochondrial ROS was measured using MitoSOX Red (mitochondrial ROS-specific fluorescent probe, Ex_510 nm_/Em_580 nm_, Invitrogen) according to the manufacturer's protocol [43]. After incubation with 5 μM MitoSOX Red for 30 min at 37°C, cells were resuspended and fluorescence intensity was measured by FCM. For fluorescence microscopy analysis, cells were incubated with 5 μM MitoSOX Red and 100 nM MitoTracker Green (label mitochondria, Ex_490 nm_/Em_516 nm_, Invitrogen) for 30 min at 37°C.

### Electrophoretic mobility shift assay (EMSA)

The EMSA probe of NF-κB binding site in the HIF-1α promoter (5′-GCGTGGGCTGGGGTGGGGACTTG CCGCCTGCGTCGC-3′) [19] was synthesized by Viagene Biotech Inc. (Jiangsu, China) and EMSA was performed using EMSA kit (Viagene) following the manufacturer's instruction [44]. Equal micrograms of nuclear proteins were mixed with the biotin-labeled NF-κB probe and poly dI:dC for 20 min at room temperature for binding reaction. Afterwards, the DNA − protein complexes were electrophoresed on a 6.5% polyacrylamide gel at 180 V for 70 min and transferred to a binding membrane. The transferred DNA was cross-linked to the membrane by ultra violet for 10 min and incubated with streptavidin conjugated horseradish peroxidase for 20 min. Reactive bands were detected by chemiluminescence reaction. NF-κB positive control was used to locate the NF-κB-probe binding band.

### Metabolism study

Levels of glucose, lactate, citrate, fumarate and ATP were measured using Glucose Colorimetric/Fluorometric Assay Kit (BioVision, Milpitas, CA, USA), Lactate Colorimetric/Fluorometric Assay Kit (BioVision), Citrate Colorimetric/Fluorometric Assay Kit, Fumarate Colorimetric Assay Kit (BioVision) and ATP Colorimetric/Fluorometric Assay Kit (BioVision) respectively following the methods provided by the manufacturer. Briefly, conditioned mediums and cell lysates were moved into upper filter devices of Amicon Ultra-0.5 10KDa spin filters (Millipore, MA, USA) and centrifuged for 20 min at 8,000*g* to be deproteinized, then the solutions in the lower centrifuge tubes were used as the samples for analysis immediately. The fresh samples filtrated from conditioned mediums were used for glucose and lactate assay, and samples from cell lysates were used for intracellular citrate, fumarate and ATP measurement by corresponding kits. The amounts of glucose uptake and lactate secretion were calculated using the concentration difference value between conditioned mediums and fresh mediums. Metabolites levels were normalized by total protein concentration of cells.

### Cell proliferation assay

Cells grew in 96-well plate were incubated with MTT (Sigma) for 4 h, then the supernatant was removed and DMSO was added to each well. After shaking the plate for 10 min, the optical density value was measured by an ELISA reader (BIO-Tek ELx800, Winooski, VT, USA) at 570 nm.

### Anchorage-independent growth assay

Anchorage-independent growth was assessed by soft agar assay and the assay was done in 6-well plates [27]. 1.5 mL complete medium containing 0.6% agar per well was solidified to form bottom layer, then 1.5 mL complete medium containing 0.3% agar and 5 × 10^3^ cells was added on the top of bottom layer. After 10 days’ incubation at 37°C, the colonies consisted of > 10 cells were counted.

### Animals study

5 weeks old male athymic nude mice were purchased from Zhejiang Chinese Medical University (Zhejiang, China). 6 × 10^6^ SGC-shCONTROL or SGC-shBECN1 cells collected in 200 μL PBS were subcutaneously injected into the back of every mouse. Once visible tumor masses established, mice in SGC-shBECN1 group were divided into two groups randomly to receive intraperitoneal injections of 0.9% NaCl or NAC (50 mg/kg) respectively thrice per week for 4 weeks. Tumor masses of all mice were measured twice a week and tumor volume was calculated using the formula: volume = (length × width^2^)/2. The mice were sacrificed when tumor volume approximately reached 200 mm^3^, then subcutaneous xenografts were harvested, fixed in 4% formaldehyde and embedded in paraffin. Immunohistological staining for BECN1, HIF-1α, E-cadherin, GLUT-1 were performed according to the procedures described earlier [41]. Liver metastatic mice models were induced by splenic injection [45]. Briefly, 5 × 10^5^ cells in 50 μL PBS were subcapsularly inoculated into spleen of each mouse. 6 weeks later, livers were excised and observed by visual inspection. When metastatic nodules in livers were discernible, the corresponding mice were regarded as liver metastatic models. All animal experiments were approved by the Animal Experimentation Committee of Zhejiang University.

### Detection of glucose uptake in subcutaneous xenograft by positron emission tomography (PET)

Fluorine-18-labeled fluorodeoxyglucose (^18^F-FDG, an analog of glucose) was synthesized using an automatic ^18^F-FDG synthesizer (FDG-F100, Sumitomo Heavy Industries, Tokyo, Japan) in the Department of Nuclear Medicine, second affiliated hospital of Zhejiang University. After fasting for 6 h, every mouse were injected with 200 μCi ^18^F-FDG through tail vein. 40 min later, mice were anesthetized and placed at the center of a microPET scanner (R4, Concorde Microsystems, Knoxville, TN, USA). Subsequently, A 10-min microPET data collection was performed. The images were reconstructed by ordered subsets expectation maximization calculating algorithm. ^18^F-FDG uptake in xenograft was calculated for the semiquantitative analysis using the formula: T/N (tumor-to-nontumor) ratio = pixel maximum uptake (percentage of injection dose) in tumor / pixel mean uptake (percentage of injection dose) in upper limb muscles.

### Patients and tissue specimens

This study consisted of 156 primary gastric cancer patients who underwent radical resection without chemotherapy and radiotherapy before surgery at the Department of Oncological Surgery in Anyang Tumor Hospital (Anyang, Henan, China) from December 2003 to December 2008. The data of patients and paraffin-embedded gastric cancer tissues were obtained from the archives of Department of Pathology in Anyang Tumor Hospital. Histological specimens of all patients were reevaluated to confirm the diagnosis by a pathologist. All patients were classified according to the 2009 criteria of the International Union Against Cancer (UICC). The study was approved by the Ethics Committee of Anyang Tumor Hospital and Anyang Hygiene Bureau.

### Tissue microarray construction

Tissue microarrays (TMAs) were constructed as previously described [46]. All donor tissues in the paraffin blocks were reviewed by a pathologist and representative areas were selected for TMA construction. Two cylindrical biopsies (1.5 mm in diameter) were taken from representative gastric cancer tissue and adjacent normal mucosa tissue of each case, and transferred to the recipient paraffin block at defined array positions. The consecutive TMA slices were used for immunohistochemical study.

### Immunohistochemical analysis and evaluation

Protein expression of BECN1, HIF-1α, E-cadherin, GLUT-1 in consecutive TMA sections were detected by immunohistochemistry. Immunohistochemical staining was evaluated and scored by two independent observers. The staining intensity of tumor cells in the whole cylinder was classified into 4 parts: negative (score 0), weak (score 1), moderate (score 2) and strong (score 3). The percentage of positive staining tumor cells was scored as follows: 0 (negative), 1 (1–25%), 2 (26–50%) and 3 (51–100%). The protein expression of every cylinder was assessed by using the sum of staining intensity score and percentage score. Next, all combining immunoreactive scores were divided into two groups: 0–3 (low protein expression group) and 4–6 (high protein expression group).

### Statistical analysis

Values are shown as means ± SEM. Student's *t*-test was performed for analyzing the comparisons of quantitative data between two groups. Chi-square or Fisher's exact tests were used to evaluate categorical data. *P* value less than 0.05 was considered statistically significant.

## SUPPLEMENTARY FIGURES


