# Supplementary material for: Inhibition of autophagy promotes metastasis and glycolysis by inducing ROS in gastric cancer cells
Source: Oncotarget. 2015 Oct 14;6(37):39839–54. doi: 10.18632/oncotarget.5674 (PMC4741864; doi:10.18632/oncotarget.5674)
Supplement: Supplementary file 1 [file oncotarget-06-39839-s001.pdf]

SUPPLEMENTARY FIGURES

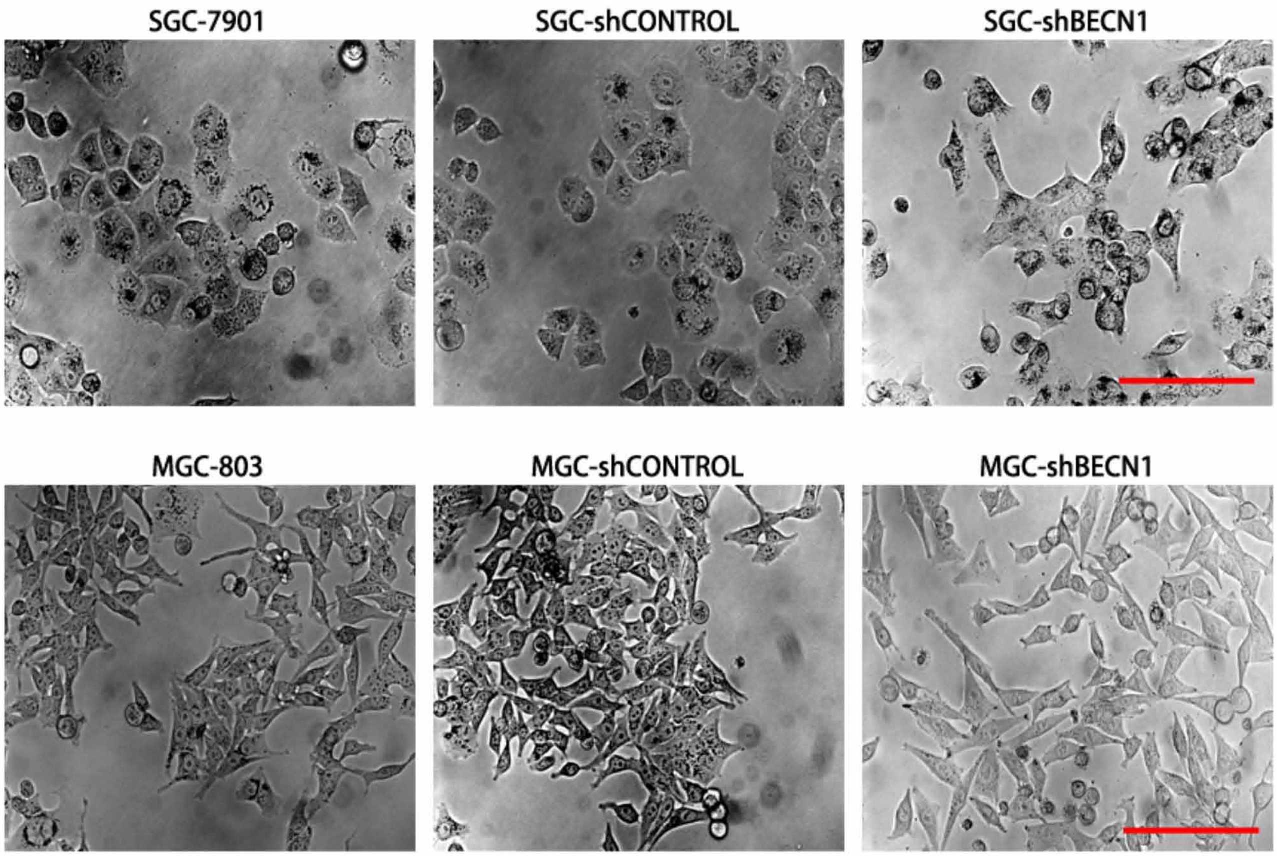

Supplementary Figure S1: Autophagy defect induces mesenchymal-like morphologies in gastric cancer cells. SGC-shBECN1 and MGC-shBECN1 cells were more spindle-like and scattered than control cells. Scale bar, 100 µm.

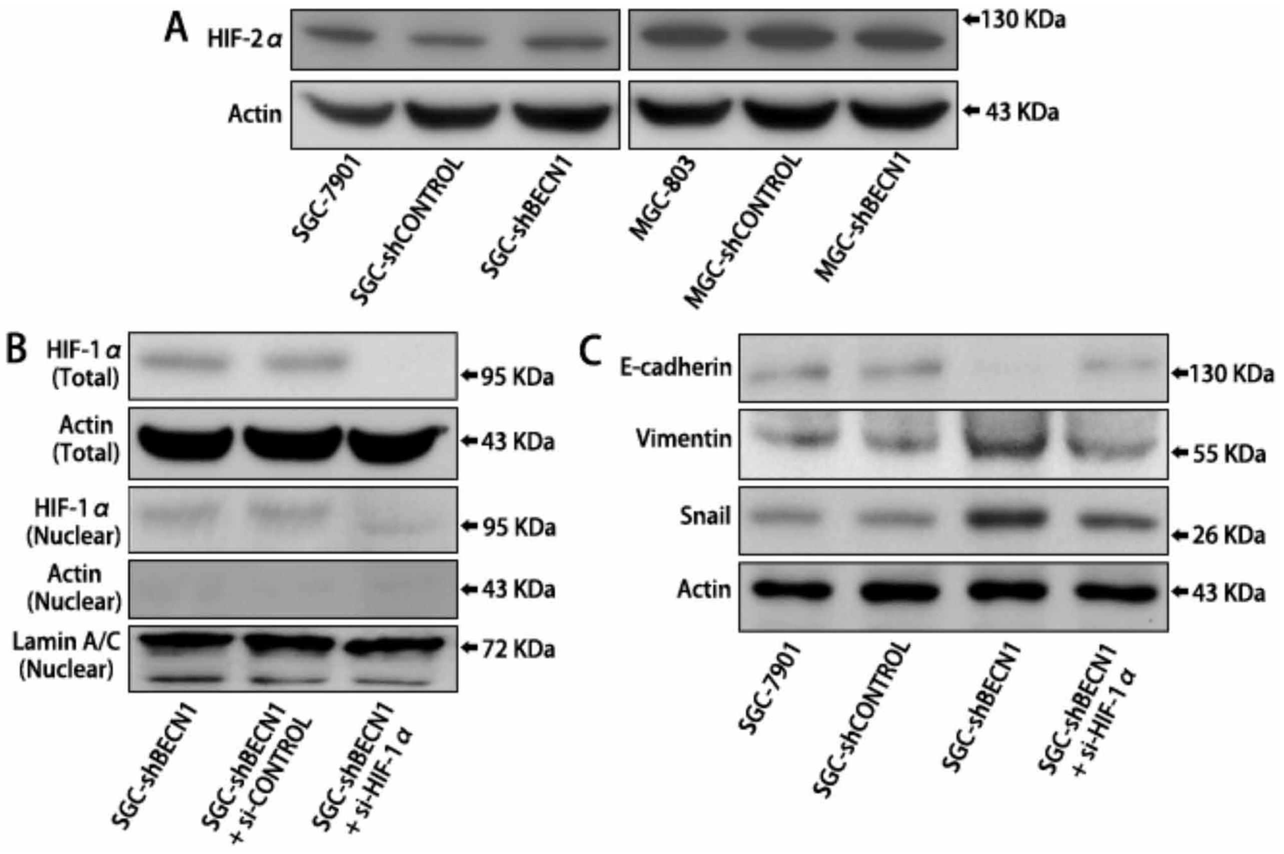

Supplementary Figure S2: Activation of HIF-1α is necessary for autophagy defect induced EMT. A. HIF-2α expression in autophagy-deficient cells and control cells were determined by Western blot. **B & C.** After transfection with HIF-1α siRNA, the expression of EMT markers in SGC-shBECN1 cells were analyzed by Western blot.

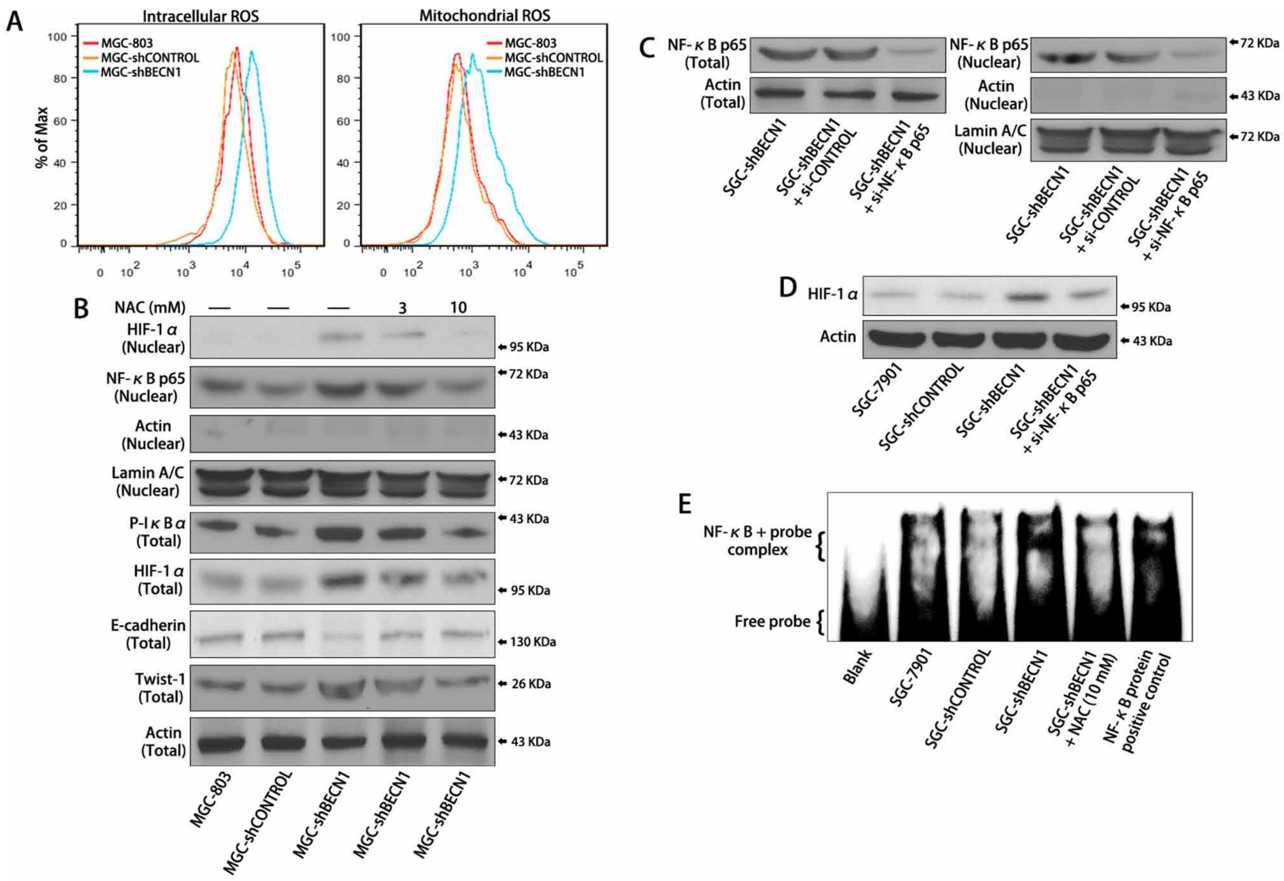

Supplementary Figure S3: Autophagy defect increases HIF-1α expression via ROS-NF-κB-HIF-1α pathway. **A.** Intracellular and mitochondrial ROS levels were determined by FCM. **B.** After treating MGC-shBECN1 cells with 3 or 10 mM NAC for 48 h, the expression of HIF-1α and NF-κB p65 in nuclei, and the expression of Phospho-IκBα, HIF-1α, E-cadherin, Twist-1 in whole cells were determined by Western blot. **C & D.** After transfection with NF-κB p65 siRNA, the HIF-1α expression in SGC-shBECN1 cells were analyzed by Western blot. **E.** After incubation with or without 10 mM NAC for 48 h, the EMSA was performed to observe the binding activity between nuclear extracts and specific probe (containing NF-κB binding site in HIF-1α promoter).

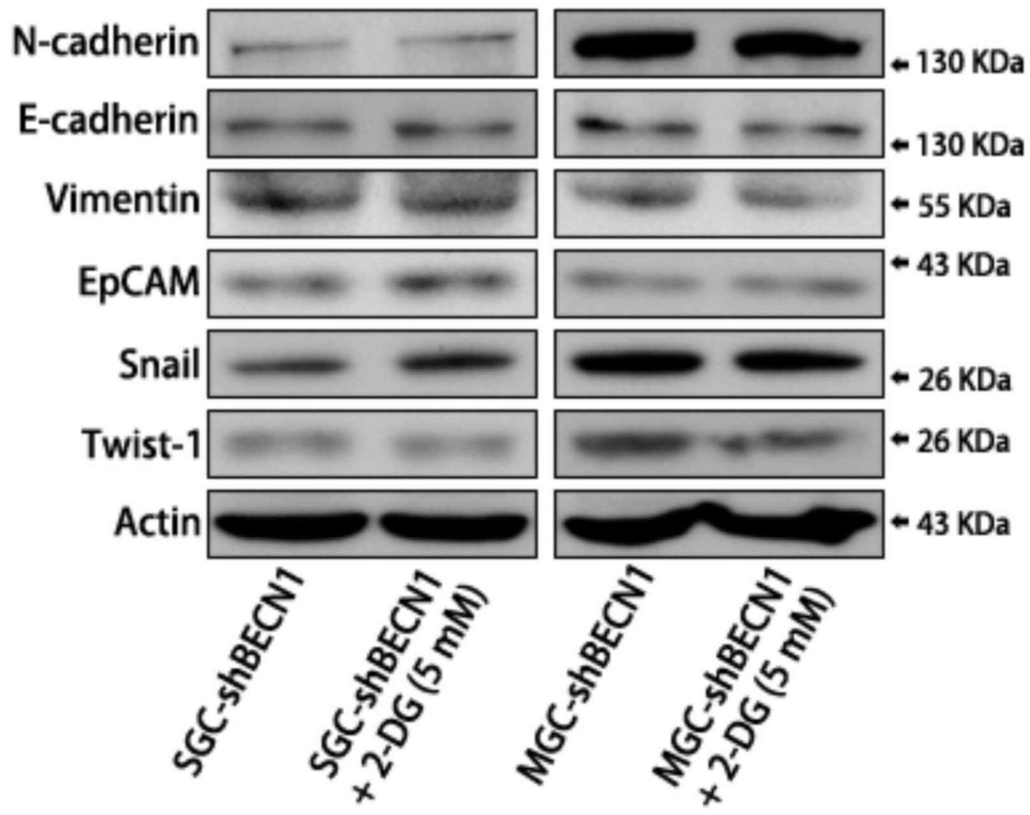

Supplementary Figure S4: Autophagy inhibition induced glycolysis has no effect on EMT. After incubation with 5 mM 2-DG for 12 h, the EMT markers in SGC-shBECN1 and MGC-shBECN1 cells were determined by Western blot.

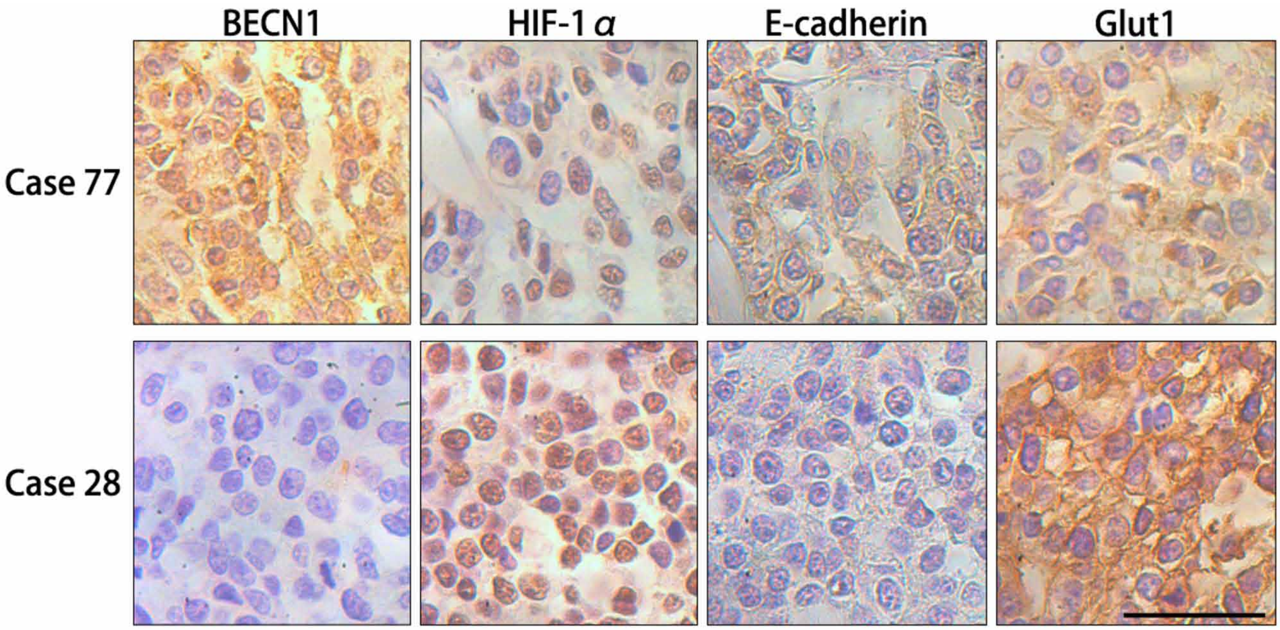

Supplementary Figure S5: Immunohistochemical analysis of consecutive sections from human gastric cancer tissues. The expression of HIF-1α, E-cadherin and Glut1 were determined in positive BECN1 expression case (case 77) and negative case (case 28) respectively. HIF-1α was mainly localized in nucleus, while Glut1 was largely distributed in cytoplasm and cytomembrane. Scale bar, 100 μm.
